# Economic burden and health-related quality of life in patients with epidermolysis bullosa in Spain

**DOI:** 10.1186/s13023-024-03328-1

**Published:** 2024-09-23

**Authors:** Isaac Aranda-Reneo, Juan Oliva-Moreno, Luz María Peña-Longobardo, Álvaro Rafael Villar-Hernández, Julio López-Bastida

**Affiliations:** 1 Economic Analysis and Finance Department, Faculty of Social Sciences and Information Technologies, University of Castilla-La Mancha, Talavera de la Reina, 45600 Spain; 2 Economic Analysis and Finance Department, Faculty of Law and Social Sciences, University of Castilla-La Mancha, Toledo, 45071 Spain; 3 ONG DEBRA España Piel de Mariposa, Málaga, 45600 Spain; 4 Faculty of Health Sciences, University of Castilla-La Mancha, Talavera de la Reina, 45600 Spain

**Keywords:** Epidermolysis bullosa (EB), Cost-of-illness, Health-related quality of life, Economic burden, Productivity loss, Informal care, Spain

## Abstract

**Background:**

. Epidermolysis bullosa (EB) is a rare genetic skin disorder characterized by fragility of skin with appearance of acute and chronic wounds. The aim of this study was to determine the economic burden and the health-related quality of life (HRQoL) of patients with epidermolysis bullosa (EB) in Spain from a societal perspective.

**Methods:**

. We conducted a cross-sectional, retrospective study including 62 patients with EB (62% dystrophic, 9.6% junctional, 3.2% Kindler syndrome, and 26% with simplex EB). Data were collected from questionnaires completed by patients or their caregivers. The costs were estimated, including not only direct healthcare costs but also direct non-healthcare costs and productivity losses. We compared severe EB (Dystrophic, Junctional EB and Kindler syndrome) to non-severe EB (simplex EB) using as reference year 2022. HRQoL was measured by generic (EQ-5D) and specific (QoLEB) questionnaires.

**Results:**

The average annual cost for an EB patient was €31,352. Direct healthcare costs represented 17.2% of the total cost, direct non-healthcare costs (mainly informal care costs) 71.3% and productivity losses 11.5% of the total cost. Participants in the severe EB group had a slightly higher average cost than participants in the non-severe EB group (€31,706 vs. €30,337). Direct healthcare costs and non-healthcare costs were higher in the severe EB group (€6,205 vs. €3,024 and €23,148 vs. €20,113) while productivity losses were higher in the non-severe EB group (€7,200 vs. €2,353). The mean utility index score, where the maximum value possible is one, was 0.45 for patients with severe EB (0.76 for their caregivers) and 0.62 for those with non-severe EB (0.77 for their caregivers).

**Conclusions:**

. The social economic burden of EB, resulting from the high direct non-healthcare cost of informal care, and from the loss of productivity, accentuates the importance of not restricting cost analysis to direct healthcare costs. This substantiates that EB, particularly severe EB represents a significant hidden cost that should be revealed to society and should be considered in the support programmes for people who suffer from this disease, and in the economic evaluation of new treatments.

**Supplementary Information:**

The online version contains supplementary material available at 10.1186/s13023-024-03328-1.

## Background

Epidermolysis Bullosa (EB) is the prototypic group of disorders with skin fragility defined by blistering from minimal mechanical trauma with disruption at the dermoepidermal junction [1]. EB can be classified into 4 main types based on the layer of the skin being affected: EB simplex (EBS), junctional EB (JEB), dystrophic EB (DEB), and Kindler EB (KEB) [2–4]. In the EU, the prevalence is estimated to be 2.4:100,000 population [5] and in the USA 11.07 per million live births [6].

The recurrence of skin blistering, or erosions has a profound impact on the quality of life of EB patients and, in the most severe forms, causes early lethality [7, 8]. About 80% of people surviving to adulthood with severe EB eventually succumb to metastatic squamous cell carcinoma originating in chronic wounds [9]. At present, there is no cure for EB [10], treatments are either symptomatic or palliative, based on the principles of good wound management and, for severe cases, on multidisciplinary care [11]. Topical agents and dressings are typically used for the treatment of skin lesions [9], and appropriate follow-up is essential to monitor the patient for a multitude of secondary psychological symptoms, in particular depression, anxiety and behavioural disturbances [3]. Thus, patients and caregivers fight daily a condition that affects the health-related quality of life (HRQoL) and force them to assign higher amounts of resources than other illnesses with access to effective treatments [3, 12, 13]. This lack of treatment leads patients and their relatives to a kind of loneliness and invisibility from public or private budgets for healthcare delivery raising the burden of this condition [14].

Very few economic impact studies have been carried out on this disease [15, 16] explaining the burden of this disease from a broad perspective. The scarce studies available show that the economic impact of EB is very high, both because of the high intensity in the use of healthcare services, and because of the high social costs that the disease imposes on patients and their affective environment [17–20]. However, there are still important gaps in information due to the small number of studies carried out [21]. In addition to the high economic costs that can be monetary values, the disease causes other intangible opportunity costs in form of loss of wellbeing as severe reduction in the HRQoL of people with EB and on their caregivers [22]. Thus, quantitative approaches are also needed since few studies have assessed HRQoL of EB patients and their caregivers.

So, this prevalence-based cost-of-illness study aims to fill this gap, examining the societal costs from a societal perspective (including healthcare and non-healthcare costs and productivity losses) and the HRQoL of patients with EB in Spain.

## Methodology

### Research design and sample

We designed a cross-sectional study of non-institutionalised patients diagnosed with EB who received outpatient care in Spain, using online recruitment methods. The Strengthening the Reporting of Observational Studies in Epidemiology (STROBE) guidelines have been followed in the study [23]. Because of the lack of patient registries at national level, subjects were recruited with the assistance of the EB association (NGO DEBRA). This patient association sent an invitation letter to all its members and to those people who had been interested in it once, introducing the study and providing a URL link through which participants could access to the study questionnaires. The criteria for eligibility were a diagnosis of EB, a non-institutionalised status and agreement to participate in the study. All patients and caregivers were informed of the study’s objectives and about data confidentiality and were asked to confirm their understanding of the study conditions and their agreement to participate. Ethical approval was obtained from the Ethical Committee of the Hospital Nuestra Señora del Prado (reference number 45/21). Participants answered the questionnaires between December 2021 and December 2022.

EB comprises a spectrum of disease-severities [1], of which DEB and JEB generally make up the most severe EB types with highest disease severity, as well as the highest prevalence of pain [14, 24], that eventually lead to premature death [25]. Because of this reason we have divided EB patients included in the study in severe EB (where we included Dystrophic, Junctional EB or Kindler EB patients) and non-severe EB (including here simplex EB patients). This classification allowed us to differentiate the population in the study by the severity of symptoms suffered according to international recommendations [1] and with local suggestions received from EB-specialised healthcare [25].

### Costing methodology

We used the prevalence approach to estimate resource use and, subsequently, the costs incurred from a societal perspective. We therefore considered all the direct healthcare resources used for prevention, treatment and rehabilitation, the other non-healthcare resources used (formal and informal care), and the productivity losses resulting from the illness within a given year. Prevalence-based cost-of-illness analysis has the advantage of incorporating measurements of total annual healthcare expenditure, which is particularly relevant for chronic conditions requiring long-term treatment, such as EB. In this context, a bottom-up costing approach was used to estimate total and average annual costs [26].

Data about resource utilisation were collected for each patient or caregiver, using specific questionnaires. To estimate resource utilisation, the questionnaires asked for details of resource use in the past twelve months. However, in order to avoid memory bias, some of these data (such as those about drug consumption, medical visits, or healthcare materials) related to the past three months or the previous month only. The questions that we included in the specific questionnaires were validated previously by specialized healthcare professionals. Productivity losses were calculated using data collected about reductions of patients’ and caregivers’ working time due to temporary and permanent sick leave or early retirement. Non-professional caregivers were also asked about informal care time. All costs were expressed in euros, 2022 being the reference year. We also collected information about education lost due to EB-related problems among participants of school age (intangible costs).

### Direct healthcare costs

The direct healthcare costs were derived from data about healthcare utilisation. Information about the number of hospital admissions, the number of emergency visits, and data about the volume of outpatient care, visits to healthcare professionals (doctors and nurses), were obtained from the questionnaires. Unit costs were obtained from the official government databases. To calculate the annual cost per patient, unit costs were multiplied by the quantities of the respective resources. Similarly, consumption of prescription drugs was obtained from the questionnaires and the unit costs of prescription drugs were obtained from the Vademecum database [27]. Finally, the costs of healthcare materials used by patients to cure blisters and other EB- related complications were estimated using the self-reported costs (out- of- pocket expenditures) of these items.

### Direct non-healthcare costs

Direct non-medical costs were quantified by aggregating two items: social care services and professional care, and informal caregivers’ time.

Formal care is non-health care provided by professional individuals or teams. This includes home help services, use of day centres, occupational centres, occupational therapy and/or training in activities of daily life, information/advice/assessment services, psychosocial care services for relatives and respite care services.

Informal care is identified as that care provided by people who are not professionally engaged in it and whose purpose is to help patients carry out their basic (ADL) and instrumental (IADL) activities of daily life [28]. Informal care time was self-reported by those people who were identified as main caregivers in the online questionnaire. They were asked about the total time spent on care in a typical day and the number of days of the week dedicated to caring for patients. Additionally, they were asked about the care time, disaggregating tasks assigned to ADL and IADL, and including the time required for monitoring and accompaniment. The main caregiver was also asked about the presence of other non-professional caregivers and about the time spent providing care. The estimations of care time in aggregate and disaggregated terms (by tasks) were compared in order to avoid inconsistencies. Likewise, as a conservative criterion, and in order to prevent joint production, we censored the time spent on caring to a maximum of 16 h per day (114 h per week) per caregiver even when the reported time spent exceeded that figure. In the sensitivity analysis, the total times reported by the main caregivers were used in the estimation. The economic assessment of care time was carried out using the replacement or proxy good method [22]. In this approach, care time is valued in terms of the costs that would be incurred if there were no possibility of providing informal care, and it was replaced by professional care. Thus the unit costs of one hour of care were obtained from the *Instituto de Mayores y Servicios Sociales* [29].

### Productivity losses

The estimation of the productivity losses of the patients was carried out using the human capital method [30]. The work time not performed was valued through the salary not received, taking this as a proxy for lost productivity. To identify lost work time, the online questionnaire was used. Here, a distinction was made between leaving the employment market because of illness (permanent disability) and temporary absences from work. Other productivity losses such as presenteeism were not addressed in the study due to the complexity of estimating them by means of an online questionnaire. Wages were adjusted by age, gender and place of residence using data from the Salary Structure Survey carried out by the National Institute of Statistics [31].

In the case of healthcare costs and formal non-healthcare costs, the health-consumer Price Index was used. In the case of informal care and labour losses, they were updated using the Statistics on Collective Labour Agreements-Ministry of Labour and Social Economy.

### Patient and caregiver outcomes

Demographic data, type of EB, and data about resource use, HRQoL and burden of care were collected from EB patients and their caregivers. The principal caregiver answered the questionnaires when the person with EB was less than 18 years old or when the patient was unable to answer the questionnaires alone. Questionnaire responses received by the research team had no identification information (i.e., name, identification, address/postcode, e-mail, or telephone). Patient and caregiver outcomes were obtained via the EQ-5D [32], the specific questionnaire QOLEB [33], and the Zarit Burden Interview [34]. The EQ-5D is a generic instrument of HRQoL commonly used in economic evaluations and routinely included in health technology assessments (HTAs). Its five dimensions (mobility, self-care, everyday activities, pain/discomfort and anxiety/depression) result in different health statuses based on the participants’ responses. These health statuses can then be used to estimate values of utilities, a score on a scale where 0 corresponds to death and 1 corresponds to perfect health, negative values being possible. These utility scores show “social tariffs” estimated using TTO methods that reveal the preferences of the general population [35]. The second part of the EQ-5D consists of a 0-100 Visual Analogue Scale (VAS), where 0 represents the worst and 100 represents the best imaginable health status. Thus, the HRQoL was defined from an empirical point of view. This allowed us to adjust how the participants’ self-reported health status affected their quality of life, estimating the utility index score. Moreover, this adjustment was made from a societal point of view since the tariff we used to calculate the utility index score associated with the self-reported health status was validated in the Spanish general population [36].

The QOLEB questionnaire [37] evaluates two elements: functional and emotional aspects. For each question there are 4 response options scored from 0 to 3 points, in which a higher score denotes a worse quality of life. The questions that relate to the functional aspects would thus score a subtotal of 0 to 36, the emotional aspects scale would score a subtotal of 0 to 15, and the total score for the questionnaire would range from 0 to a maximum of 51 points. The impact revealed by this global score could then be classified as follows, according to the score range achieved: very slight (0–4 points); slight (5–9 points); moderate (10–19 points); severe (20–34 points); and very severe (35–51 points) [33, 37–39].

Finally, we used the Zarit Burden Interview (the 22-item version) to measure the subjective burden among caregivers. Each item is a statement to which the caregiver is asked to respond using a 5-point scale, with options ranging from 0 (never) to 4 (nearly always). The total score ranges from 0 to 88, where scores under 21 correspond to little or no burden, and scores over 61 represent a severe burden [34].

### Statistics analysis

We carried out a descriptive analysis of the cost variables by type of EB and by age. All types of EB were included in the study, but we compared the most severe EB types (severe EB group) with EB simplex (non-severe EB group). We also explored the differences according to age since adult patients have more skin, so more blisters are expected. Cost outcomes were continuous, so we used mean and standard deviation (SD) to summarise these data by EB group (severe EB versus non-severe EB). We analysed differences between groups using t-test assuming unequal variances and considering several scenarios for statistical significance, 90% when p-value was lower than 0.1, 95% when p-value was lower than 0.05 and 99% when p-value was lower than 0.01.

Health-related quality of life, as utility index scores or VAS results from EQ-5D tools was also shown, using mean and SD by group of EB disease (severe EB versus non-severe EB). We also used t-test with unequal variances to assess differences between EB groups, and the same scenarios to assess the statistical significance used in the costs analysis. Since we included the proxy version of EQ-5D-3 L to assess the HRQoL of paediatric patients and patients unable to answer the online questionnaires, we applied several criteria to use the HRQoL data. We excluded from the HRQoL analysis those participants who provided the same VAS score on the EQ-5D-5 L (as caregiver) and on the EQ-5D-5 L (as the proxy respondent) together with those who did not complete the health dimensions and VAS that the EQ-5D tool contains. All analyses were carried out using STATA software.

## Results

An invitation letter to participate in the study was sent to 226 EB patients receiving 101 answers (44% response rate). However, only 62 questionaries included valid data, so we analysed data collected from 62 individuals diagnosed with EB (Table 1). The average patient’s age was 25.86 (SD = 23.87), and 51% were female. The average caregiver’s age was 47.4 (SD = 11.2) and 71% were women. Most of the patients had primary education (36%), were married (64%) and were on permanent sick leave (42%). With regard to the type of EB, 62% of the individuals had dystrophic EB (DEB), 26% of them had EB simplex (EBS), 9.6% junctional EB (JEB) and 3.2% Kindler EB (KEB). 95% of the caregivers were married or had a partner, and 62% of them were the mother or father of the patient with EB.

**Table 1:** Characteristics of participants in the study by group

	Severe EB (*N* = 46)	Non-severe EB (*N* = 16)	All (*N* = 62)
**Patients**			
**Age**,** mean (SD)**	24.00 (22.83)	31.00 (26.65)	25.86 (23.87)
**Female**,** %**	55.56	37.50	50.82
**Highest level of education %**			
No education	0	0	4.00
Primary school	25.00	41.18	36.00
Secondary school	62.50	17.65	32.00
University	12.50	41.18	28.00
**Employment status**,** %**			
Employed	11.11	12.50	11.54
Unemployed	11.11	0	7.69
Temporary sick leave	5.56	0	3.85
Permanent sick leave	38.89	50.00	42.31
Retired	11.11	25.00	15.38
Other	22.22	12.50	19.23
**Marital status**,** %**			
Single	35.29	25.00	32.00
Married	64.71	62.50	64.00
Separated	0	12.50	4.00
**Caregivers**	**Severe EB**(*N* = 16)	**Non-severe EB**(*N* = 5)	**All** (*N* = 21)
**Age**,** mean (SD)**	48.0 (12.7)	45.4 (4.3)	47.4 (11.2)
**Female**,** %**	81.3	40.0	71.4
**Marital status: married/ partner (%)**	93.3	100.0	95.0
**Relationship with person cared for (%)**			
Partner	18.8	20.0	19.1
Son/Daughter	12.5	20.0	14.3
Mother/Father	62.5	60.0	61.9
Other	6.2	0.0	4.8

Non-Severe EB group included participants with EBS; Severe EB group included participants with JEB, DEB and KEB

Estimated average annual cost per person was €31,352. Direct non-healthcare costs made up the largest proportion, at 71.33% of the total average cost per person, followed by direct healthcare costs, at 17.17% of the total average cost per person, and loss of productivity (11.49%). The most important category of healthcare costs was the specialist visit, which reached €1,795 (5.72% of the total cost), followed by nursing cures, which represented €743 per year (2.37% of the total cost). The most important category of direct non-healthcare costs was informal care, with an average cost of €22,197 (70.79% of total costs), a high proportion of which was related to main caregivers (55.11% of total costs) (Table 2).

**Table 2:** Average annual costs per patient by EB group (€2022)

	Severe EB (*N* = 46)	Non-severe EB (*N* = 16)	All (*N* = 62)	*p*-value
Drugs	218.40 (516.10)	36.08 (65.57)	171.35 (451.68)	0.17
Medical tests	471.66 (1,073.31)	585.87 (1,783.31)	501.14 (1,278.43)	0.76
Healthcare materials^†^	602.96 (1,483.35)	1,364 (3,118.55)	799.16 (2,031.61)	0.2
Health professionals’ visits	435.08 (613.08)	119.77 (328.25)	353.71 (568.44)	0.76
Specialist visits	2,157.99 (2,611.4)	754.13 (1,229.43)	1,795.70 (2,405.39)	0.04^**^
Nursing Cures	1,002.74 (4,908.14)	0	743.96 (4,238.73)	0.42
Hospitalization	700.79 (1,670.26)	24.58 (98.34)	526.29 (1,466.08)	0.11
Emergency visits	133.62 (332.08)	47.88 (108.52)	111.49 (292.71)	0.32
Surgical interventions	144.06 (344.38)	31.21 (124.87)	114.94 (306.27)	0.21
Transport	337.91 (732.48)	60 (162.15)	266.19 (645.98)	0.14
**Total healthcare cost**	**6**,**204.98 (7**,**748.86)**	**3**,**023.55 (4**,**144.72)**	**5**,**383.97 (7**,**105.58)**	0.12
**Informal care main caregiver**	18,280.43 (32,702.04)	14,400.74 (30,793.14)	17,279.22 (32,015.87)	0.68
**Informal care other caregivers**	4,851.004 (14,649.89)	5,112.685 (18,773.18)	4,918.534 (15,652.56)	0.95
**Informal care total**	23,131.44 (39,457.29)	19,513.43 (44,612.12)	22,197.76 (40,502.68)	0.76
Formal care	0	556.59 (2,226.37)	143.63 (1,131.01)	0.09^*^
Social services	16.79 (77.68)	42.62 (170.5)	23.46 (108.30)	0.42
**Total non-healthcare cost**	**23**,**148.23 (39**,**472.64)**	**20**,**112.65 (44**,**396.42)**	**22**,**364.85 (40**,**446.09)**	0.80
**Productivity losses**	2,353.23 (7,406.31)	7,200.48 (12,507.64	3,604.13 (9,138.23)	0.07^*^
**Total cost**	**31**,**706.44 (43**,**463.59)**	**30**,**336.68 (43**,**748.38)**	**31**,**352.96 (43**,**180.88)**	0.91

Non-Severe EB group included participants with EBS; Severe EB group included participants with JEB, DEB and KS. *Significant at 90%. ** Significant at 95%. ^†^Health materials included out-of-pocket payments made by patients; †† Nurse cures included only time provided by specialized-EB nurse who carried out cures, health materials used were not included

With regard to the costs incurred by EB simplex patients (non-severe patients group), the mean annual costs were only €1,016.28 less than the mean annual costs of all types of EB. Direct healthcare costs were €3,023, direct non-healthcare costs were €20,112 and loss of productivity costs were €7,200 per patient. In the severe EB group (junctional EB, dystrophic EB, and Kindler EB), mean annual costs were €31,706, direct healthcare costs were €6,204, direct non-healthcare costs were €23,148 and loss of productivity costs were €2,353 per patient. However, we did not find notable differences in mean annual costs which depended on the severity of the disease. Nevertheless, it can be noticed that patients in the severe EB group used more drugs, paid more visits to health professionals (such as specialist doctors and specialist nurses), needed more emergency attention, had more hospital admissions, and needed more informal caregiving time than the non-severe EB group (see Table A1 in additional file 1. Besides, when comparing the annual costs according to the time spent on curing wounds (less than one hour compared to more than one), it appears notable and statistically significant differences in all costs (see Table A3 in additional file 1).

**Table 3:** Average annual costs per patient by age (€2022)

	Children (*N* = 36)	Adults (*N* = 24)	*p*-value
Drugs	248.66 (577)	60.85 (105.01)	0.12
Medical tests	125.90 (322.84)	1,041.07 (1,895.4)	< 0,01^***^
Healthcare materials	1,080.66 (2,535.20)	430.16 (928.58)	0.23
Health professionals’ visits	193.53 (417.16)	611.41 (690.72)	< 0,01^***^
Specialist visits	2,083.23 (2,889.73)	1,438.72 (1,495.10)	0.32
Nursing Cures	800.8 (4,804.8)	0	0.42
Hospitalization	397.82 (1,217.05)	762.84 (1,827.08)	0.36
Emergency visits	79.52 (179.10)	168.75 (415.11)	0.26
Surgical interventions	25.72 (154.33)	258.35 (421.27)	< 0,01^***^
Transport	286.66 (759.53)	232.66 (468.08)	0.76
**Total hc cost**	**5**,**322.54 (7**,**677.99)**	**5**,**004.85 (5**,**537.52)**	0.86
**Informal care main**	22,693.65 (36,318.9)	7,845.57 (21,356.94)	0.08^*^
**Informal care other**	6,178.85 (17,762.24)	3,437.94 (12,759.62)	0.52
**Informal care total**	28,872.5 (45,591.21)	11,283.51 (29,811.53)	0.1
Formal care	0	371.06 (1,817.83)	0.22
Social services	0	60.60 (169.59)	0.04^**^
**Total non-hc cost**	**28**,**872.5 (45**,**591.21)**	**11**,**715.18 (29**,**770.62)**	0.11
**Productivity losses**	-	9,310.68 (12,885.41)	< 0,01^***^
**Total cost**	**34**,**195.04 (47**,**356.49)**	**26**,**030.71 (36**,**158.41)**	0.48

Children: those participants younger than 18 years old. Note: *significant at 90%. ** Significant at 95%. ***Significant at 99%. There were two missing values in the age information

We found that the cost for children was notably higher (€34,195 vs. €26,031), although the differences were not statistically significant. In terms of healthcare costs, the differences between those for children and those for adults were very small (€317.69 higher for children). But we found notable differences in non-healthcare costs, where we observed a greater need for informal non-health care for children than for adults (€28,873 vs. €11,284) and no productivity losses, since children cannot work (Table 3). However, among 30 children identified with EB, there was an average of 20.9 schooldays lost per year due the disease. Distinguishing by type of EB, those with severe EB lost a total of 24.4 schooldays, while those with simple EB lost 3.4 schooldays. It should be noted that there were two cases in which children indicated a large number of schooldays lost (250 and 150).

The HRQoL analysis was reduced to only 39 participants (63%) who answered the EQ-5D questionnaires (24 using the proxy version of EQ-5D-3 L and 15 the self-reported EQ-5D-5 L), and 19 participants (31%) who completed the QoLEB. The HRQoL of the patients and caregivers was assessed using the utility index score that can be estimated using the “time trade-off” (TTO) social tariff proposed by the EuroQoL group, as well as the VAS included in the Eq. 5D instrument. The EQ-5D-5 L social tariff estimated a utility of 0.61 (SD = 0.2) for the patients with EB included in the study, while the EQ-5D-5 L VAS produced a score of 48.7 (SD = 21.33). Meanwhile, the proxy EQ-5D-3 L social tariff showed a score of 0.5 (SD = 0.36), while the EQ-5D-3 L VAS produced a score of 65.6 (SD = 15.84). So, there was a higher utility index score from self-reported health status than from the proxy version, but a lower score on the VAS. However, when analysing differences between EB groups, self-reported HRQoL reached a higher utility index score but a lower VAS score in the severe EB group (0.61 and 51 from self-reported versus 0.45 and 64.24 from the proxy HRQoL) and lower utility index score and VAS score in the non-severe EB group (0.58 and 35 from self-reported compared to 0.62 and 69 from proxy) (see Figure A1 from the additional file 2). Similar figures were observed in HRQoL when comparing by the time taken to cure EB-related wounds. Those patients who need more than one hour to heal their wounds stated higher VAS but lower utility scores. However, those patients who need more time to cure their wounds presented worse HRQoL (see Table A3 from the additional file 2).

This high impact of EB on HRQoL observed with the EQ-5D instruments was confirmed with the EB-specific QOL tool, QoLEB. According to this instrument, 11% of the patients suffered a “very severe impact” on the quality of life (14% in the severe EB group and 0% in the non-severe EB group), 26% a “severe impact” (29% in the severe EB group and 20% in the non-severe EB group), 53% a “moderate impact” (43% in the severe EB group and 80% in the non-severe EB group) and 11% a “slight impact” (14% in the severe EB group and 0% in the non-severe EB group). This impact mainly affected functional aspects, and differences, although not statistically significant, were observed between groups (3.41 points lower in the non-severe EB group, which means a higher health-related quality of life in the functional dimensions). We observed a similar impact among groups in the emotional dimension (7.2 points in the non-severe EB group versus 6.3 in the severe EB group) (see Table A1 and Table A2 in the additional file 2).

For caregivers, the social tariff score rose to 0.76 (SD = 0.15) and the EQ-5D-VAS score was 54.13 (SD = 15.71). In contrast with the self-reported HRQoL of patients, the caregivers of participants in the severe EB group reported a lower HRQoL than caregivers of participants in the non-severe EB group (see Figure A4 on the additional file 2). Anxiety/depression-related conditions were the main reason for lower HRQoL among caregivers, and ‘pain/discomfort’ was the second most frequent reason (see Figure A5 in the additional file 2). Finally, the burden for caregivers was mild, as the average Zarit Burden Interview score was 32.6 (SD = 12.8) and non-significant differences in the burden of care were observed between groups, according to the Zarit results (32.4, SD = 14.2 in the severe EB group compared to 33.5, SD = 4.8 in the non-severe EB group).

## Discussion

Epidermolysis bullosa is a disease that requires an enormous mobilisation of healthcare and social resources to meet the needs of people suffering from this disease. The economic burden of EB was estimated at an annual cost which, on average, exceeds €30,000 per patient, with medical consultations and care materials being the main items of healthcare expenditure. From a societal perspective, the most relevant cost was that of non-professional care (informal care) provided by families.

This study has therefore tried to provide comprehensive information about the costs of EB and adds to the existing literature about the costs of EB and the impact on HRQoL. This would reflect the high degree of non-healthcare needs of a patient with EB, regardless of the degree of severity of the disease.

When comparing the results obtained for EB patients, it is observed that patients with EB have higher annual costs than patients with other chronic illnesses such as human immunodeficiency virus (HIV), acquired immunodeficiency syndrome (AIDS) (€13,823) [40] and stroke survivors during the first year after the stroke (€ 13,826) [41–43]. In addition, the costs of EB are very similar to, or higher than, those of patients with other rare diseases such as Cystic fibrosis, Prader-Willi syndrome, haemophilia, Duchenne muscular dystrophy, Fragile X syndrome, scleroderma, juvenile idiopathic arthritis, histiocytosis, ataxia and TGCT (tenosynovial giant-cell tumour) [41–43]. Although the degree of severity is associated with higher health care and informal care costs, this difference is not statistically significant. On the contrary, the (unexpected) result that productivity losses are higher in non-severe patients should be studied and confirmed in future studies of the disease.

The BURQOL project, which studied the costs of EB in 8 European countries, including Spain, was published in 2016 [17]. Subsequently, Angelis et al. 2022 extracted a subsample of patients (with dystrophic EB) from the BURQOL project and updated the costs to 2020 [44]. While there are differences in healthcare costs between these two papers and our findings, it can be explained by the different time points at which the studies were conducted (2011–2013 vs. 2021–2022) and by the specific type of EB selected by Angelis et al. [44]. However, the largest differences are identified in the results of non-health costs, which are lower in our work. The main reason for these differences is that, for Spain, the percentage of patients who received informal care is higher in the work of Angelis et al. 2016 [17] and significantly higher in the work of Angelis et al. 2022 [44]. Since there are no official records to identify in which study the estimates of informal care provision are closer to the reality of the population of patients with EB, our results can be interpreted as a conservative estimate of non-health care costs. Knowing what percentage of patients in total, by type of EB, require professional and non-professional (informal) non-health care, as well as knowing the met and unmet needs of patients, is a challenge that will require future research.

We found a low HRQoL in caregivers according to the utility and VAS scores, which could be the result of a real, but not perceived, caregiving burden, since the main caregivers had low Zarit index values, which denotes an absence of high caregiver-perceived burden. So, our findings open up several hypotheses to be tested in future studies that will have to do with whether the Zarit instrument is a valid tool for revealing caregiver burden in the case of EB and diseases with similar characteristics. Specific instruments should be developed to reveal the burden borne by these caregivers, as well as studies that attempt to delve deeper into the causes of the differences between real and perceived caregiver burden, and that help to identify the elements that are associated with losses in the HRQoL of caregivers.

However, the HRQoL of people who have EB is much lower than the HRQoL of the general population for the same age (0.92) [45]. In fact, it is also lower than that of people who experience chronic illnesses such as HIV/AIDS (0.78), digestive diseases (0.74), Diabetes Mellitus (0.69), heart problems (0.69), respiratory tract disease (0.71), degenerative osteoarthritis (0.68), back pain (0.73), osteoporosis (0.63), or anxiety/depression (0.66), and similar to that of people with other rare diseases such as TGCT, ataxia, cystic fibrosis, Prader-Willi syndrome, haemophilia, epidermolysis bullosa, Fragile X syndrome, scleroderma, juvenile idiopathic arthritis, or histiocytosis [40–43, 45, 46].

When comparing the HRQoL results with other research that used the QoLEB instrument we observed worse HRQoL in the non-severe EB group (18.6, SD = 3.6) compared to Spanish (11.8, SD = 6.4) [33] or Australian (13.7,SD = 8.7) patients but similar to US patients (19, IQR = 5–30) [21]. However, we observed similar HRQoL in the severe EB group comparting to recessive dystrophic epidermolysis bullosa in Brazilian patients (20.2, SD = 9.2) or Spanish patients (20.7, SD = 10.6) [33] and better HRQoL compared to recessive dystrophic epidermolysis bullosa Australian patients (35.5, SD = 12.7) [21]. We found similar distribution in the functional/emotional aspects compared with other international studies [21].

It should be noted that the estimated total cost figure should be interpreted as a conservative value since some of the costs, such as those due to schooldays lost, are intangible and were not converted into monetary units. On the other hand, our healthcare costs analysis may have underestimated the costs, since it was not possible to include the costs of healthcare materials provided in healthcare centres (publicly funded in Spain). The Servei Calatá de la Salut (publicly funded healthcare provider) provided us with an estimate of the cost of the healthcare material delivered [47] to EB patients, and this figure increased the €10,000 per patient in 2022. Moreover, we should consider other limitations in our study: (i) although the sample was almost evenly distributed among degrees of severity, we could not guarantee the avoidance of the selection bias that occurs in most studies with low sample sizes, (ii) we tested the questionnaires with healthcare professionals to avoid recall bias, but some participants could not perfectly remember the resource consumption, (iii) we applied an inclusion criterion to avoid inconsistences in the caregiver health-related quality of life questionnaires when patients answered instead of the caregiver. So the low sample size made us to recommend caution when extrapolating our results to EB patients’ caregivers. Finally, we decided to classify participants according to the main EB forms (EBS, EBD, JEB and KEB) into two groups: severe (those with EBD, JEB and KEB) and non-severe (EBS). However, this classification could not be entirely accurate since EBS patients can suffer from very severe symptoms, and some JEB and EBD patients show rather ‘mild’ symptoms [1, 48].

In future studies, the ideal approach for addressing the societal costs of EB should include combined access to information from patients’ medical records, together with collaboration agreements with healthcare providers to assess all the resources used, and also specific questionnaires aimed at patients and caregivers, such as those used in this study, which delve deeper into those aspects that fall outside the healthcare sphere and belong more to the family and social sphere of patients and caregivers.

## Conclusion

EB involves considerable societal costs, including very high economic costs and a deterioration in the HRQoL of both patients and their informal caregivers. The societal economic burden of EB, shared between the high direct non-healthcare costs resulting from the use of informal care, and from the loss of productivity, accentuates the importance of not restricting cost analysis to direct healthcare costs. EB represents a significant hidden cost that should be revealed to society and should be considered in the support programmes for people who suffer from this disease and in the economic evaluation of new treatments.

## Electronic supplementary material

Below is the link to the electronic supplementary material.

Supplementary Material 1

## Data Availability

The datasets generated and/or analysed during the current study are not publicly available due privacy restriction included in the informed consent signed by the study participants.

## Declarations

This study was conducted in accordance with the Declaration of Helsinki. The protocol was approved by the hospitals’ ethics committees in accordance with national and regional regulations.
