# Supplementary material for: Economic burden and health-related quality of life in patients with epidermolysis bullosa in Spain
Source: Orphanet J Rare Dis. 2024 Sep 23;19:352. doi: 10.1186/s13023-024-03328-1 (PMC11421099; doi:10.1186/s13023-024-03328-1)
Supplement: Supplementary file 1 — Supplementary Material 1 [file 13023_2024_3328_MOESM1_ESM.docx]

Additional file 2: Health-related Quality of Life (detailed)

Figure A1: Patients’ health-related quality of life by type of EB, proxy-reported (left) and self-reported (right)

Non-Severe EB group included participants with EBS; Severe EB group included participants with JEB, DEB and KS

Figure A2. Description of Patients’ HRQoL using EQ5D-5L (self-reported) by EB group

Non-Severe EB group included participants with EBS; Severe EB group included participants with JEB, DEB and KS.

Figure A2. Description of Patients’ HRQoL using EQ5D-3L (proxy-reported) by EB group

Non-Severe EB group included participants with EBS; Severe EB group included participants with JEB, DEB and KS.

Table A1. Quality of life summary results by QoLEB^(a)^ instrument.

|  | All sample (n=19) | SEVERE EB (n=14) | NON-SEVERE EB (n=5) | Difference | p-value |
| --- | --- | --- | --- | --- | --- |
| Functional (0-36) | 12.32 (6,95) | 13.2 (7,7) | 9.8 (3,77) | -3.41 | 0.22 |
| Emotional (0-15) | 6.53 (2,12) | 6.3 (2,3) | 7.2 (1,64) | 0.91 | 0.36 |
| Total (0-51) | 20.21 (8,88) | 20.8 (10,2) | 18.6 (3,58) | -2.19 | 0.50 |

(a) higher score denotes worse quality of life. Non-Severe EB group included participants with EBS; Severe EB group included participants with JEB, DEB and KS.

Table A2. Impact on quality of life by EB group of patients according to QoLEB.

|  | All sample (n=19) | Severe EB (n=14) | Non-Severe EB (n=5) |
| --- | --- | --- | --- |
| Very slight (0-4) | 0% | 0% | 0% |
| Slight (5-9) | 11% | 14% | 0% |
| Moderate (10-19) | 53% | 43% | 80% |
| Severe (20-34) | 26% | 29% | 20% |
| Very severe (35-51) | 11% | 14% | 0% |

Non-Severe EB group included participants with EBS; Severe EB group included participants with JEB, DEB and KS.

Table A3: Patients’ health-related quality of life according to the time taken on curing wounds, mean (SD)

|  | LOWER THAN 1 HOUR TO CURE (n= 9) | MORE THAN 1 HOUR TO CURE (n= 5) | p-value |
| --- | --- | --- | --- |
| EQ5D5L^a^-VAS | 43,78 (18,63) | 57,6 (25,13) | 0,32 |
| EQ5D5L^a^-utility | 0,64 (0,03) | 0,52 (0,14) | 0,29 |
|  | **LOWER THAN 1 HOUR TO CURE (n=6)** | **MORE THAN 1 HOUR TO CURE (n=18)** | **p-value** |
| EQ5D3L^b^-VAS | 72,66 (11,72) | 63,28 (16,61) | 0,15 |
| EQ5D3L^b^-utility score | 0,66 (0,14) | 0,45 (0,08) | 0,22 |
|  | **LOWER THAN 1 HOUR TO CURE (n= 12)** | **MORE THAN 1 HOUR TO CURE (n= 7)** | **p-value** |
| QoLEB ^c^ | 16,42 (1,28) | 26,71 (4,18) | 0,05 |

^a^ Self-reported health-related quality of life; ^b^ proxy-reported health-related quality of life; ^c^ higher values indicate worse quality of life.

Figure A4. Caregiver's HRQoL using the EQ5D by EB group.

Non-Severe EB group included participants with EBS; Severe EB group included participants with JEB, DEB and KS

Figure A5. Descriptive caregivers’ EQ5D dimensions.

Non-Severe EB group included participants with EBS; Severe EB group included participants with JEB, DEB and KS.
